# Placental accommodations for transport and metabolism during intra-uterine crowding in pigs

**DOI:** 10.1186/2049-1891-5-55

**Published:** 2014-12-15

**Authors:** Jeffrey L Vallet, Anthony K McNeel, Jeremy R Miles, Bradley A Freking

**Affiliations:** U.S. Department of Agriculture, Agricultural Research Service, U.S. Meat Animal Research Center, State Spur 18D, Clay Center, NE 68933 USA

**Keywords:** Glycosaminoglycans, Nutrient transport, Placenta

## Abstract

Litter size and birth weights are limited by uterine capacity, defined as the ability of the uterus to maintain the appropriate development of some number of conceptuses. Uterine capacity is the result of the combined effects of uterine, placental and embryo/fetal function. The number of living conceptuses that the uterus is capable of supporting is greater during early gestation compared to later gestation. Plots of log fetal weight versus log placental weight also indicate that fetal weights are less sensitive to reduced placental weight (and therefore reduced intrauterine space) in early gestation compared to late gestation. However, even in late gestation, mechanisms still exist that maintain fetal growth when the size of the placenta is reduced. One such mechanism is likely to be improved development of the folded placental-epithelial/maternal-epithelial bilayer. Fold depth, and therefore the maternal fetal interactive surface, increases as gestation advances and is greater in placenta from small fetuses. On the fetal side of the placenta, the epithelial bilayer is embedded in stromal tissue. Glycosaminoglycans are major components of stroma, including hyaluronan and heparan sulfate. Hyaluronidases and heparanases are present within placental tissues, and likely play roles in modification of stromal components to facilitate fold development. Glycosaminoglycans are polymers of forms of glucose (glucosamine, glucuronic acid, iduronic acid) suggesting that glycosaminoglycan synthesis may compete with the glucose needs of the developing fetus. Pig conceptuses are fructogenic, such that a substantial portion of glucose transferred from mother to fetus is converted to fructose. Fructose is an intermediate product in the synthesis of glucosamine from glucose, and glucosamine is linked to regulation of trophoblast cell proliferation through regulation of mTOR. These findings suggest a link between glucose, fructose, glucosamine synthesis, GAG production, and placental morphogenesis, but the details of these interactions remain unclear. In addition, recent placental epithelial transcriptome analysis identified several glucose, amino acid, lipid, vitamin, mineral and hormone transporter mechanisms within the placenta. Further elucidation of mechanisms of placental morphogenesis and solute transport could provide clues to improving nutrient transport to the pig fetus, potentially increasing litter size and piglet birth weights.

**Electronic supplementary material:**

The online version of this article (doi:10.1186/2049-1891-5-55) contains supplementary material, which is available to authorized users.

## Introduction

A recent analysis by the National Pork Board indicated that between 2007 and 2012, the number of piglets born alive per litter improved by 0.25 piglets per year, while the number of piglets weaned per litter improved by only 0.16 piglets per year [1]. This difference translates into an increase in preweaning mortality of 0.3% per year, and is a concern not only from a production standpoint, but also from an animal well-being standpoint. The increase in the number born alive is likely due to a combination of improved management and genetic selection of breeding animals. Although it was not measured in the study, the disparity between the improvement in the number of piglets born alive and the number of piglets weaned is most likely due to the well known depression in piglet birth weight associated with increased litter size [2]. Reduced birth weights are a primary factor associated with preweaning mortality [3–5], and may explain the observed increase in preweaning mortality. Finally, depression of birth weights with increasing litter size is a manifestation of limitations on conceptus development that are imposed by uterine capacity.

### Uterine capacity

Uterine capacity has been defined in a variety of ways. The number of piglets that can be supported by the uterus during gestation until farrowing if the number of potential fetuses is not limiting, is a common definition [6]. This definition includes stillborn piglets, which die during farrowing, but does not include mummified fetuses, which die at some point during gestation. It does not fully describe uterine capacity in totality because it does not include any component reflecting differences in piglet birth weights. Clearly, a pig uterus that can support ten 2 kg fetuses has greater uterine capacity than a pig uterus that can support ten 1 kg fetuses. Finally, in practice, the independence of uterine capacity from the number of potential fetuses is also problematic. The relationship between the maximum litter size that can be maintained by the uterus and the number of potential embryos is almost certain to be curvilinear. Reports suggest that at moderate intrauterine crowding, litter size reaches a peak. Significant further crowding beyond this point reduces the number of viable embryos/fetuses, probably by reducing the number of embryos able to obtain sufficient uterine space for survival due to intrauterine competition for space among embryos [7].

Studies of intrauterine crowding and uterine capacity have employed a variety of methods. The most straightforward to apply is unilateral hysterectomy-ovariectomy (UHO) [8]. In this surgical method, one ovary and one uterine horn are removed. Compensatory ovarian hypertrophy results in a normal ovulation rate, with only half the available uterine space. The intrauterine crowding that results is relatively moderate and uniform between pigs compared to that caused by superovulation [9], and the UHO surgical procedure is simpler and more reproducible than embryo transfer [10]. The surgery can be done early in life (i.e., before puberty, typically 100 to 160 days of age) and the pig can be allowed to recover, reducing the interference of the surgery with normal pregnancy physiology that occurs when uterine ligation methods are used [11]. Genetic selection also increases uterine crowding [12], but takes several generations to accomplish. Litter size in UHO females has been reported to be independent of ovulation rate, and it has been assumed that the litter size obtained is half the uterine capacity [8] for that animal. The UHO procedure has been used successfully to select pigs for uterine capacity [13, 14].

### Placental efficiency

There have been some approaches to placental efficiency that have not required an understanding of the underlying mechanisms, but their utility has been controversial and limited. One approach, the fetal weight to placental weight ratio, has been suggested as a broad indicator of placental efficiency [15, 16]. An early report that this ratio could be used to select animals for placental efficiency, with subsequent improvements in litter size [17], was not confirmed using more rigorous selection methods over several generations [18]. Also, litter size was improved in a line selected for uterine capacity but selection did not alter the fetal weight to placental weight ratio and selection for ovulation rate did not alter litter size but did alter the fetal weight to placental weight ratio [19]. Although it makes sense that the size of a fetus supported by a given size of placenta should reflect placental efficiency, use of the ratio as a measure of placental efficiency fails to consider the ability of the fetus and placenta to adjust efficiency as the size of the placenta is reduced. In other words, if compensatory mechanisms exist to adjust placental efficiency as the size of the placenta is reduced, the fetal weight to placental weight ratio does not measure absolute placental efficiency for a given conceptus, as would be required to implement genetic selection. Instead, the fetal weight to placental weight ratio measures placental efficiency for that conceptus given the size of the placenta. If that same conceptus developed a smaller placenta, compensatory mechanisms would raise placental efficiency, which would then be reflected in the fetal weight to placental weight ratio.

**Figure 1:**
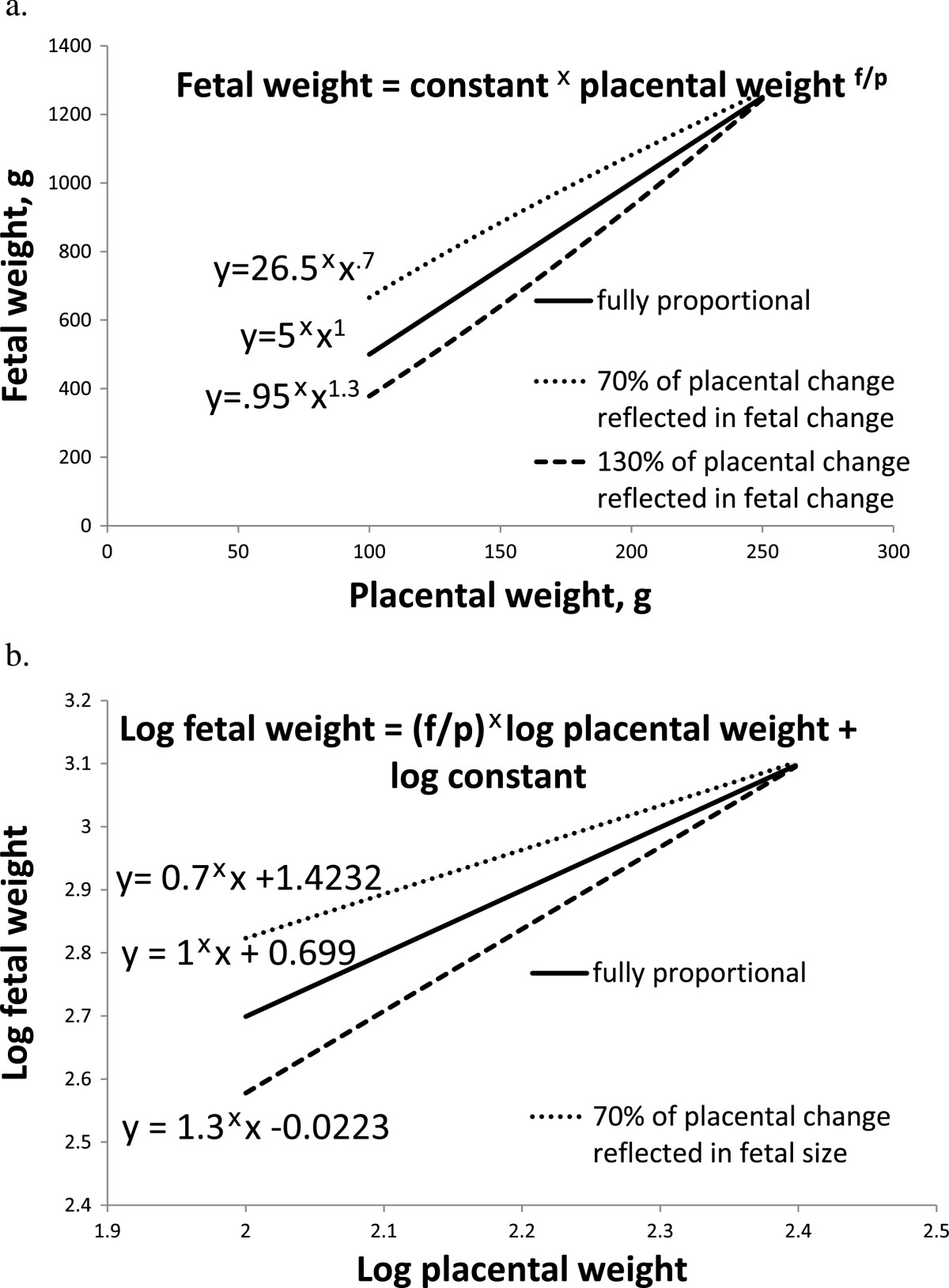
**Possible allometric relationships between fetal weight and placental weight are illustrated.** According to Huxley [22], the relationship between fetal weight and placental weight can be described with the equation fetal weight = constant × placental weight^f/p^ where f and p represent growth rates of the fetus and placenta, respectively. Given this relationship, the slope of the linear relationship between log fetal weight and log placental weight is f/p, the relative growth rates of the two components. The graphs represent **(a)** untransformed and **(b)** log transformed hypothetical relationships where growth is fully proportional (i.e., growth rates are equal; solid line), only 70% of changes in placental weight are reflected in changes in fetal weight (a fetal sparing effect where fetal growth is relatively insensitive to differences in placental weight; small dashes), and 130% of changes in placental weight are reflected in changes in fetal weight (fetal growth is highly sensitive to differences in placental weight; large dashes). In pigs, even during late gestation, the slope of the log fetal weight to log placental weight relationship is less than one, indicating fetal sparing [21].

Evidence of compensatory mechanisms for placental efficiency can be found by examining relationships between log fetal weight and log placental weight during gestation [20, 21]. Huxley [22] proposed that the slope of log-log relationships between individual body parts or between a body part and the entire organism reflects the relative growth between the two (Figure 1). A slope of 1 indicates proportional growth, greater than 1 indicates that the y variable grows faster than the x variable, less than one indicates that the y variable grows more slowly than the x variable [23]. Extending this concept to the fetus and placenta, a slope of one indicates a fully proportional relationship, the fetus is proportionally larger if the placenta is larger. As placental size is reduced, fetal size is also reduced, and the ratio of the two is the same (placental efficiency is constant and no compensatory mechanisms are present). A slope less than one indicates fetal growth is not fully proportional to the size of the placenta. As placental size is reduced, fetal size is less affected (placental efficiency increases suggesting compensatory mechanisms are present). The slope of the relationship between log fetal weight and log placental weight increases throughout gestation, but even in late gestation is still less than one [21]. Growth of the fetus is not very dependent on placental size during early gestation, and becomes more dependent as gestation advances, but is never fully dependent on placental size. These relationships indicate that compensatory mechanisms are present, even in late gestation.

### Factors affecting placental function

The pig placenta is classified as diffuse epitheliochorial [24]. This distinguishes it from other livestock species in that there are no placentomes (making it diffuse), and both the fetal and maternal epithelial cell layers are maintained throughout gestation (making it epitheliochorial). Numerous factors affect the efficiency of placental transport of nutrients. Generally, these factors fall into two broad categories, physical and nutrient specific. Physical factors include maternal and fetal blood flow (both rates and physical arrangement relative to one another), fetal and maternal interactive surface area, and the distance between maternal and fetal capillaries. These factors globally affect the transport of all nutrients. Nutrient specific factors are as varied as the nutrients transported. To fully understand placental transport, it is necessary to fully characterize the contribution of both categories.

**Figure 2:**
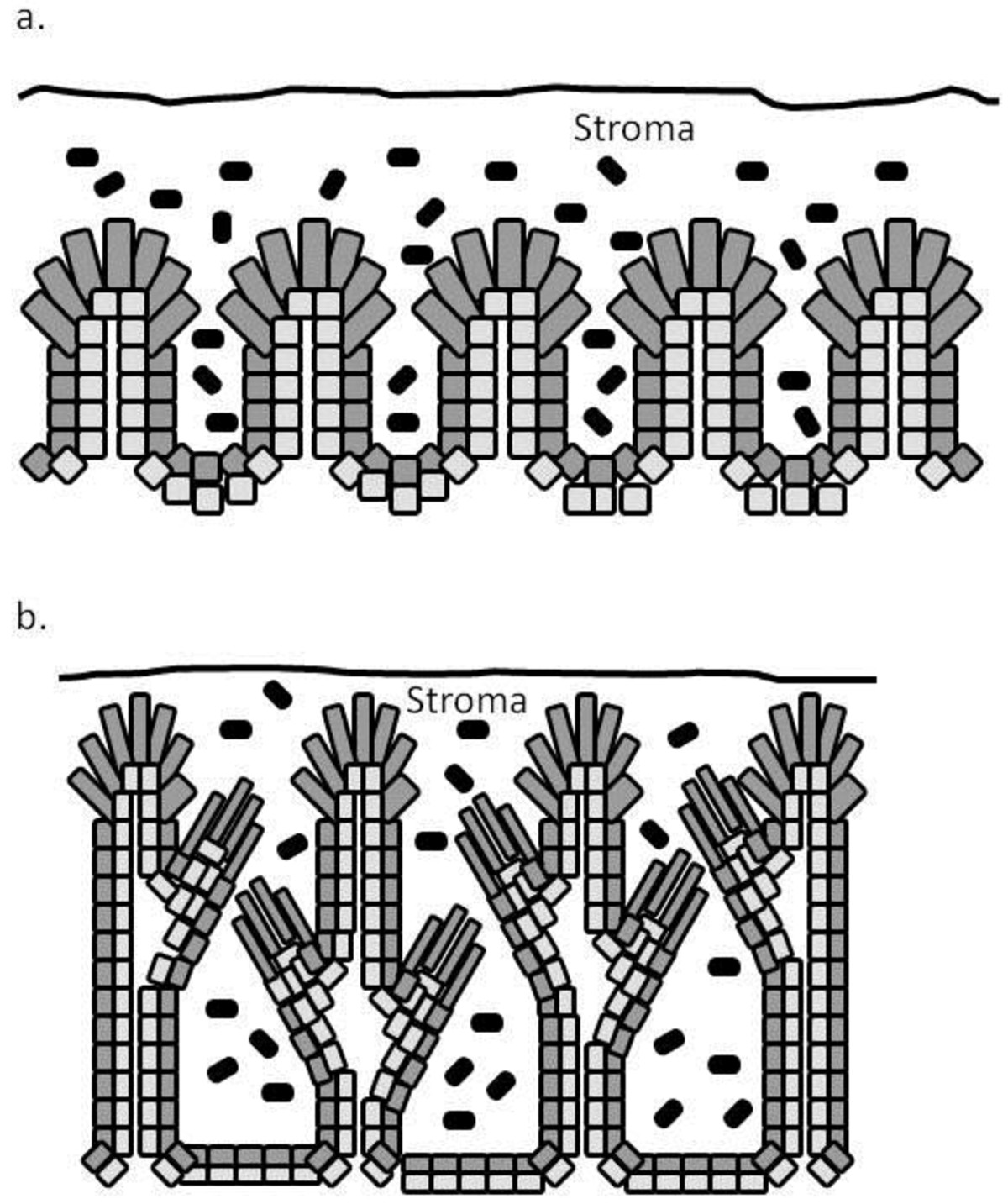
**Schematic showing changes in the pig placental epithelial bilayer during gestation.** On day 60 of pregnancy **(a)** the folded bilayer has a regular appearance and is embedded in placental stroma. The stromal covering of the folds is relatively deep. The fetal placental epithelial cells at the tops (fetus side is up in the figures) of the folds are tall columnar in appearance, the sides and bottoms are lined with cubiodal epithelia. By day 105 of gestation **(b)**, the folded bilayer is wider, more complex and has secondary folds, which increases the interacting surface area. The epithelial cells of the bilayer are thinner to reduce the distance solutes must diffuse. The stromal space between the folds is expanded to accommodate the secondary folds. The stroma covering the folds is thinner and can be absent in placenta of small fetuses. Fetal and maternal capillaries (not shown) are immediately adjacent to the epithelial cells within the folded structure and are arranged in a countercurrent fashion.

The physical structure of the placenta divides into areolae and interareolar areas [25]. Areolae take up histotroph secreted by uterine glands [26, 27]. Histotroph represents nutrient specific transport and will be dealt with later. The interareolar areas consist of a maternal endometrial epithelium layer tightly adhered to a fetal epithelium layer (trophoblast), which becomes folded beginning about day 30 to 35 of gestation (Figure 2). The folds become more elaborate and more extensive as gestation advances [20, 28]. During mid gestation, the folds are relatively close to one another. During late gestation, fetal placental stroma grows into the region between the folds creating a stromal space between folds, and secondary folds develop into this space. As this is taking place, the bilayer separating maternal and fetal capillaries becomes thinner, to the point that maternal and fetal capillaries actually indent into, but do not penetrate, the epithelial cell layers. The indentation of capillaries reduces the distance between maternal and fetal capillaries to as little as 2 microns [28].

Blood flow and angiogenesis of the pig uterus and placenta have received a great deal of attention. Increased uterine blood flow in response to increased litter size has been reported, but appears to be limited [29, 30]. The fetal weight to placental weight ratio has been reported to be correlated with placental blood vessel density in a comparison of Meishan and European breed pigs [15]. These results are difficult to interpret, primarily because it is not clear whether the blood vessels adjacent to the bilayer were measured preferentially or whether all placental vessels were included. Because the epithelial bilayer is the interactive surface between the dam and fetus and solute diffusion decreases rapidly with increased distance, only the capillaries directly adjacent to the bilayer are relevant to nutrient transport. Finally, placental expression of vascular endothelial growth factor (VEGF), a known angiogenic growth factor, has been reported to increase with advancing gestation and is also correlated with the fetal weight to placental weight ratio [31, 32]. These results suggest that fetal placental angiogenesis may contribute to placental efficiency.

Leiser and Dantzer [33] used microcast techniques to visualize maternal and fetal capillaries and concluded that they were arranged in a cross-countercurrent fashion on opposite sides of and directly adjacent to the epithelial bilayer. According to Leiser and Dantzer [33], maternal blood enters the structure at the top of the folds and exits at the bottom (fetal side of the placenta is “up”), fetal blood enters near the bottom of the folds and exits at the top. Thus, the capillary arrangement exchanges solutes between the dam and fetus within the axis perpendicular to the surface of the placenta. The distance of the interacting surface along this axis (the width of the folds) should affect efficiency of exchange, because a greater width would increase the interaction distance between the two blood supplies and facilitate exchange.

Vallet and Freking [20] measured this width throughout gestation and compared placenta associated with the largest and smallest fetuses in litters from UHO gilts. Fold width increased during late gestation, and was greater in the placenta of small fetuses compared to large fetuses. In the same experiment, the width of the stromal area between the top of the folds and the allantois was also measured. This region may represent further room for the width of the folds to expand. The width of this stromal region became progressively less with advancing gestation, and was less in the placenta of small fetuses compared to large fetuses. In placenta of some of the smallest fetuses, a stromal region above the folds was absent, suggesting that no further expansion of the folded bilayer was possible.

Given these results, we have hypothesized that expansion of the width of the folds represents a compensatory mechanism for increasing placental efficiency if the size of the placenta is restricted (e.g., during intrauterine crowding). If expansion of the folds exceeds the available placental stromal width, further compensation is not possible and fetal growth and development are impaired. If this impairment is severe enough, the fetus is lost, contributing to late gestation loss that occurs during intrauterine crowding.

If this hypothesis is true, one strategy for improving litter size would be to focus on mechanisms of placental stroma and folded bilayer development. Stromal tissues are made up of fibroblasts embedded in extracellular matrix, large constituents of which are glycosaminoglycans like hyaluronan and heparan sulfate [34, 35]. Glycosaminoglycans are polymers of sugar [36, 37], probably originating primarily from glucose transported from dam to fetus. Thus, adequate stromal development, and therefore ultimately placental compensatory development, is likely to be dependent on adequate glucose transport. Logically, this suggests that placental development and fetal development compete for glucose resources. This is consistent with reports in sheep where the placenta consumes 60% of incoming glucose [38]. This competition is likely to be most severe during late gestation and suggests there may be mechanisms that regulate whether glucose is directed to either fetal or placental development.

One such mechanism might be generation of fructose. Livestock conceptuses are fructogenic [39], meaning that a substantial portion of incoming glucose is converted to fructose, primarily by the placenta [40]. Studies indicate that fructose is oxidized to CO_2_ at 20% the rate of glucose, so once glucose is converted to fructose it is relatively less available for oxidation [41, 42]. Fructose is an intermediate in glucosamine synthesis and glucosamine is needed for glycosaminoglycan production [43]. Also of interest, conversion of fructose to glucosamine stimulates mTOR and regulates cell proliferation in porcine and human trophoblast cells, tying fructose to placental development [44, 45]. We recently compared glucose and fructose concentrations in the blood of fetuses from UHO gilts during late gestation. Neither sugar was related to fetal weight despite large differences (weight range 423 to 1473 g). Glucose concentrations were positively correlated with placental weight (*P* <0.05; weight range 73 to 413 g), but fructose concentrations were not (Vallet, unpublished observations). This suggests that the concentration of fructose is likely to be regulated within the conceptus. Whether fructose is synthesized to sequester it from oxidation and preserve its use for glycosaminoglycan synthesis, or for some other role in metabolism, requires further study.

Factors that control folded bilayer development are largely unknown. Two types of placental trophoblast cells are present within the folded bilayer, tall columnar cells at the tops of the folds and cuboidal cells at the bottom and sides of the folds. The location of the tall columnar cells at the top of the folds suggested that they might play a role in widening of the folded structure by erosion of the surrounding stroma. To begin to address the control of fold development, enzymes that degrade extracellular matrix components of the stroma, which would be needed to alter bilayer structure, were examined in the placenta throughout gestation. The cDNAs corresponding to two forms of hyaluronidase were cloned from placental tissue. Similarly, two molecular weight forms of hyaluronidase were detectable in placental tissue extracts using zymography [35], but the correspondence between the cDNAs and molecular weight forms of the proteins has not been fully clarified. Both protein forms increased with advancing gestation and were greater in placenta from small fetuses, which is consistent with a role for these enzymes in fold formation. In addition to hyaluronidase, heparanase was also cloned from placental tissues and expression was examined using in situ hybridization [46]. Heparanase mRNA was localized to trophoblast cells lining the sides and bottom of the folded bilayer, no labeling was observed in trophoblast cells at the tops of the folds. Heparanase plays a role in modifications of the basement membrane during cell movements within tissues [47]. Thus, contrary to our hypothesis, the heparanase results suggest that modifications of the folded bilayer occur along the sides and bottoms of the folds.

To gain further insight into the role of the two trophoblast cell types in fold development, we performed a transcriptomic analysis of the two cell types that had been collected from average weight fetuses on day 85 of gestation [48] using laser capture microdissection combined with high-throughput Illumina sequencing of cDNA (gestation length in the pig is 114 days). Expression levels of 7413 genes were observed by the two cell types combined. Increased expression of 434 genes were observed for tall columnar trophoblast cells compared to cuboidal trophoblast cells, while the expression of 1088 genes were greater in cuboidal trophoblast cells compared to tall columnar cells. Ingenuity Pathway Analysis was used to determine biological pathways that would be affected by these changes in transcription. Of relevance to fold development, pathways involved in cell invasion, motility and movement were increased in the cuboidal cells lining the sides and bottom of the folds compared to tall columnar cells at the tops of the folds. Heparanase sequences were observed in this analysis, and preferential transcription of heparanase in cuboidal cells compared to tall columnar cells was confirmed. These results again suggest that fold development, or at least modifications of the morphology of the epithelial bilayer, is controlled by changes in the sides and bottom of the folds.

### Nutrient specific mechanisms

Most nutrients transported from the dam to the fetus have specific mechanisms that facilitate transfer. Oxygen and CO_2_ cross the bilayer by simple diffusion, but hemoglobin [49] and carbonic anhydrase [50] facilitate transport, respectively. Glucose and amino acids are polar molecules that do not cross cell membranes easily, and various proteins facilitate their uptake by cells. Very little is known regarding the specifics of lipid transport. One report indicated that transport of fatty acid across the pig placenta is very poor [51]. Vitamin and mineral transport is facilitated in a variety of ways. For example, iron in the form of uteroferrin is secreted by the uterine glands as a component of histotroph, which is then taken up by placental epithelial cells within the areolae by pinocytosis [26]. Histotroph is likely to be a vehicle for the transfer of a variety of nutrients in a similar fashion. An example of a nutrient specific mechanism that facilitates vitamin transport are placental folate binding proteins [52, 53], which likely mediate folate transport to the developing conceptus.

Our transcriptomic analysis of placental trophoblast cells presented an opportunity to survey genes involved in nutrient transport. To gain further information on transport mechanisms, we focused attention on solute carrier (SLC) genes expressed by the two different types of trophoblast cells. Swine genome 9.2, the version of the genome used for matching of transcriptomic sequences, included 239 SLC genes. This only included SLC genes placed in the completed sequence, and does not include all known SLC genes (e.g., notably absent are SLC2 sugar transporter genes SLC2A1, 2, 3, 5, 6, 9 and 11). In addition to this limitation, only genes with average expression between the two cell types of greater than 1.8 fragments per kilobase of exon model mapped per million mapped reads (FPKM) were considered to be present in the cells [48] (Table 1). Nevertheless, the results provide information on an extensive list of SLC genes expressed by trophoblast cells, and indicate that numerous genes for glucose, amino acid, lipid, vitamin and mineral transport proteins are expressed by placental trophoblast cells.

**Table 1:** **Expression (FPKM) of SLC genes by short cuboidal and tall columnar trophoblast epithelial cells (see Figure** 2 **) from pig placenta collected on day 85 of gestation by laser capture microdissection (n = 4 pigs)**

Gene	Substrate	Name	P value SC versus TC	Short cuboidal	Tall columnar
SLC1A3	Amino Acid	solute carrier family 1 (glial high affinity glutamate transporter), member 3	0.008342	103.518	152.46*
SLC1A5	Amino Acid	solute carrier family 1 (neutral amino acid transporter), member 5	0.005574	19.865	29.593*
SLC2A10	Glucose	solute carrier family 2 (facilitated glucose transporter), member 10	0.356081	1.774	2.437
SLC2A12	Glucose	solute carrier family 2 (facilitated glucose transporter), member 12	0.019601	93.214	124.979*
SLC2A13	Inositol	solute carrier family 2 (facilitated glucose transporter), member 13	0.012995	38.491*	26.879
SLC2A4	Glucose	solute carrier family 2 (facilitated glucose transporter), member 4	0.04111	3.39*	2.185
SLC3A1	Amino Acid	solute carrier family 3 (cystine, dibasic and neutral amino acid transporters, activator of cystine, dibasic and neutral amino acid transport), member 1	0.676755	29.255	28.814
SLC4A1AP	None	solute carrier family 4 (anion exchanger), member 1, adaptor protein	0.510953	3.625	3.356
SLC4A4	Bicarbonate	solute carrier family 4, sodium bicarbonate cotransporter, member 4	0.40478	3.657	2.806
SLC4A7	Bicarbonate	solute carrier family 4, sodium bicarbonate cotransporter, member 7	0.0625	3.787	2.531
SLC5A6	Vitamins	solute carrier family 5 (sodium-dependent vitamin transporter), member 6	2.08E-05	27.352*	7.827
SLC6A6	Amino Acid	solute carrier family 6 (neurotransmitter transporter, taurine), member 6	0.700481	3.691	4.132
SLC6A8	Creatine	solute carrier family 6 (neurotransmitter transporter, creatine), member 8	0.0925	3.97	2.598
SLC7A2	Amino Acid	solute carrier family 7 (cationic amino acid transporter, y + system), member 2	0.35208	2.194	1.559
SLC7A4	Amino Acid?	solute carrier family 7 (orphan transporter), member 4	0.612879	101.015	99.302
SLC7A7	Amino Acid	solute carrier family 7 (amino acid transporter light chain, y + L system), member 7	0.048306	3.179*	2.013
SLC7A8	Amino Acid	solute carrier family 7 (amino acid transporter light chain, L system), member 8	0.092703	6.23	4.956
SLC7A9	Amino Acid	solute carrier family 7 (glycoprotein-associated amino acid transporter light chain, bo,+ system), member 9	0.004643	2.419*	4.289
SLC9A1	Na+/H+	solute carrier family 9, subfamily A (NHE1, cation proton antiporter 1), member 1	0.462724	2.827	3.147
SLC9A3R1	Na+/H+	solute carrier family 9, subfamily A (NHE3, cation proton antiporter 3), member 3 regulator 1	4.51E-05	22.04*	10.738
SLC9A6	Na+/H+	solute carrier family 9, subfamily A (NHE6, cation proton antiporter 6), member 6	0.577227	3.025	3.173
SLC9A8	Na+/H+	solute carrier family 9, subfamily A (NHE8, cation proton antiporter 8), member 8	0.195039	5.92	4.937
SLC9A9	Na+/H+	solute carrier family 9, subfamily A (NHE9, cation proton antiporter 9), member 9	0.109026	4.589	6.648
SLC10A7	steroids	solute carrier family 10 (sodium/bile acid cotransporter family), member 7	0.226002	2.569	3.55
SLC12A6	K+/Cl-	solute carrier family 12 (potassium/chloride transporters), member 6	0.717437	6.458	6.382
SLC13A3	Dicarboxylate	solute carrier family 13 (sodium-dependent dicarboxylate transporter), member 3	0.004193	19.292*	28.657
SLC13A4	Sulfate	solute carrier family 13 (sodium/sulfate symporters), member 4	1.73E-06	91.288*	44.882
SLC14A1	Urea	solute carrier family 14 (urea transporter), member 1 (Kidd blood group)	0.579099	3.759	5.211
SLC15A1	Di/tripeptides	solute carrier family 15 (oligopeptide transporter), member 1	0.10901	2.251	1.715
SLC15A2	Di/tripeptides	solute carrier family 15 (H+/peptide transporter), member 2	6.49E-05	84.452	147.72*
SLC16A10	Amino Acid	solute carrier family 16, member 10 (aromatic amino acid transporter)	0.057011	47.966	38.846
SLC16A12	Creatine	solute carrier family 16, member 12 (monocarboxylic acid transporter 12)	5.09E-05	7.95*	2.177
SLC16A14	?	solute carrier family 16, member 14 (monocarboxylic acid transporter 14)	0.002365	9.309*	5.651
SLC16A7	Lactate	solute carrier family 16, member 7 (monocarboxylic acid transporter 2)	0.01361	3.627*	1.939
SLC16A9	Urate	solute carrier family 16, member 9 (monocarboxylic acid transporter 9)	0.348855	3.476	2.655
SLC17A5	Acid sugars	solute carrier family 17 (anion/sugar transporter), member 5	0.627423	7.041	7.408
SLC19A2	Thiamine	solute carrier family 19 (thiamine transporter), member 2	0.009728	3.455*	2.193
SLC20A1	Phosphate	solute carrier family 20 (phosphate transporter), member 1	0.003578	8.612*	4.872
SLC22A18	Organic cations	solute carrier family 22, member 18	0.12071	2.883	1.365
SLC22A23	?	solute carrier family 22, member 23	0.396248	3.315	3.06
SLC22A3	Polyamines	solute carrier family 22 (extraneuronal monoamine transporter), member 3	0.000145	3.563*	0.81
SLC25A1	Mitochondrial citrate, malate	solute carrier family 25 (mitochondrial carrier; citrate transporter), member 1	0.347209	20.091	17.097
SLC25A11	Mitochondrial oxoglutarate, malate	solute carrier family 25 (mitochondrial carrier; oxoglutarate carrier), member 11	0.108779	5.333	3.636
SLC25A12	Mitochondrial Amino Acid	solute carrier family 25 (aspartate/glutamate carrier), member 12	0.131405	7.194	5.996
SLC25A13	Mitochondrial Amino Acid	solute carrier family 25 (aspartate/glutamate carrier), member 13	0.409356	17.13	15.642
SLC25A14	Mitochondrial H+ (uncoupling)	solute carrier family 25 (mitochondrial carrier, brain), member 14	0.706134	1.957	2.11
SLC25A17	Mitochondrial CoA, FAD	solute carrier family 25 (mitochondrial carrier; peroxisomal membrane protein, 34 kDa), member 17	0.721955	7.931	8.946
SLC25A20	Mitochondrial carnitine	solute carrier family 25 (carnitine/acylcarnitine translocase), member 20	0.536736	18.412	20.059
SLC25A24	Mitochondrial ATP	solute carrier family 25 (mitochondrial carrier; phosphate carrier), member 24	0.345305	12.014	10.704
SLC25A25	Mitochondrial ATP	solute carrier family 25 (mitochondrial carrier; phosphate carrier), member 25	0.219067	1.837	2.319
SLC25A26	Mitochondrial adenosyl-methionine	solute carrier family 25 (S-adenosylmethionine carrier), member 26	0.458853	1.883	1.809
SLC25A27	Mitochondrial H+ (uncoupling)	solute carrier family 25, member 27	0.050934	3.482*	2.227
SLC25A28	Mitochondrial iron	solute carrier family 25 (mitochondrial iron transporter), member 28	0.398812	2.266	1.939
SLC25A3	Mitochondrial phosphate	solute carrier family 25 (mitochondrial carrier; phosphate carrier), member 3	0.465844	207.118	195.026
SLC25A32	Mitochondrial folate	solute carrier family 25 (mitochondrial folate carrier), member 32	0.342663	2.325	3.289
SLC25A33	Mitochondrial UTP	solute carrier family 25 (pyrimidine nucleotide carrier), member 33	0.02715	3.591*	1.889
SLC25A36	Mitochondrial UTP	solute carrier family 25 (pyrimidine nucleotide carrier), member 36	0.479823	10.419	11.013
SLC25A37	Mitochondrial iron	solute carrier family 25 (mitochondrial iron transporter), member 37	0.684822	8.405	8.581
SLC25A43	Mitochondrial ?	solute carrier family 25, member 43	0.004424	2.411	4.746*
SLC25A44	Mitochondrial ?	solute carrier family 25, member 44	0.660881	4.946	5.013
SLC25A46	Mitochondrial ?	solute carrier family 25, member 46	0.714232	3.622	4.053
SLC25A5	Mitochondrial ADP, ATP	solute carrier family 25 (mitochondrial carrier; adenine nucleotide translocator), member 5	0.225204	73.01	94.084
SLC26A2	Sulfate, chloride, hydroxyl ions	solute carrier family 26 (sulfate transporter), member 2	0.002566	18.742*	9.657
SLC27A4	Long chain fatty acids	solute carrier family 27 (fatty acid transporter), member 4	0.718866	2.528	2.328
SLC27A6	Long chain fatty acids	solute carrier family 27 (fatty acid transporter), member 6	0.001938	5.536*	1.159
SLC29A1	Nucleosides	solute carrier family 29 (nucleoside transporters), member 1	0.002403	51.871*	28.705
SLC29A3	Nucleosides	solute carrier family 29 (nucleoside transporters), member 3	0.0282	4.818*	3.052
SLC30A4	Zinc	solute carrier family 30 (zinc transporter), member 4	0.137271	2.308	1.538
SLC30A6	Zinc	solute carrier family 30 (zinc transporter), member 6	0.544857	6.784	6.425
SLC30A7	Zinc	solute carrier family 30 (zinc transporter), member 7	0.606204	10.946	11.388
SLC30A9	Zinc	solute carrier family 30 (zinc transporter), member 9	0.040343	12.638*	9.474
SLC31A2	Copper	solute carrier family 31 (copper transporters), member 2	0.692963	3.737	4.636
SLC35A1	Nucleotide sugar	solute carrier family 35 (CMP-sialic acid transporter), member A1	0.001966	8.876*	3.703
SLC35A3	Nucleotide sugar?	solute carrier family 35 (UDP-N-acetylglucosamine (UDP-GlcNAc) transporter), member A3	0.003442	19.107	33.186*
SLC35A4	Nucleotide sugar?	solute carrier family 35, member A4	0.717081	2.659	2.795
SLC35A5	Nucleotide sugar?	solute carrier family 35, member A5	0.683471	5.015	5.377
SLC35B1	Adenosine phospho-sulfate	solute carrier family 35, member B1	0.347209	14.398	13.503
SLC35B2	Adenosine phospho-sulfate	solute carrier family 35, member B2	0.53865	2.75	2.625
SLC35B3	Adenosine phospho-sulfate	solute carrier family 35, member B3	0.353606	6.322	5.239
SLC35C1	Nucleotide sugar	solute carrier family 35, member C1	0.00016	19.1*	6.88
SLC35D1	Nucleotide sugar	solute carrier family 35 (UDP-glucuronic acid/UDP-N-acetylgalactosamine dual transporter), member D1	0.688317	3.286	2.87
SLC35E1	?	solute carrier family 35, member E1	0.066081	4.953	2.826
SLC35E3	?	solute carrier family 35, member E3	0.265752	2.049	1.612
SLC35F2	?	solute carrier family 35, member F2	0.10158	3.188	2.186
SLC35F5	?	solute carrier family 35, member F5	0.289527	22.192	24.888
SLC36A1	Amino acid	solute carrier family 36 (proton/amino acid symporter), member 1	0.157349	19.844	16.848
SLC36A4	Amino acid	solute carrier family 36 (proton/amino acid symporter), member 4	0.159179	2.836	1.761
SLC37A1	Sugar phosphate	solute carrier family 37 (glycerol-3-phosphate transporter), member 1	0.000295	3.492*	1.626
SLC37A4	Sugar phosphate	solute carrier family 37 (glucose-6-phosphate transporter), member 4	0.196418	6.082	5.027
SLC38A1	Amino acid	solute carrier family 38, member 1	0.023371	11.839*	9.529
SLC38A6	Amino acid?	solute carrier family 38, member 6	0.00099	3.834	7.593*
SLC38A7	Glutamine	solute carrier family 38, member 7	0.657535	2.284	2.435
SLC38A9	Amino acid?	solute carrier family 38, member 9	0.203752	4.997	3.792
SLC39A11	Zinc	solute carrier family 39 (metal ion transporter), member 11	0.519439	18.918	20.158
SLC39A14	Zinc, iron, cadmium	solute carrier family 39 (zinc transporter), member 14	5.13E-05	19.797*	8.718
SLC39A7	Zinc	solute carrier family 39 (zinc transporter), member 7	0.644212	9.946	10.299
SLC39A8	Zinc, cadmium	solute carrier family 39 (zinc transporter), member 8	0.219082	6.948	4.375
SLC39A9	Zinc	solute carrier family 39 (zinc transporter), member 9	0.029289	12.607*	8.939
SLC44A1	Choline	solute carrier family 44, member 1	0.004045	29.547	45.151*
SLC44A2	Choline	solute carrier family 44, member 2	0.502802	22.687	21.741
SLC44A3	Choline?	solute carrier family 44, member 3	0.014683	6.273	10.268*
SLC44A4	Thiamine pyro-phosphate, choline	solute carrier family 44, member 4	0.002671	6.012*	2.58
SLC46A3	Folate?	solute carrier family 46, member 3	0.208452	4.484	3.561
SLC47A1	Organic cations (e.g. creatinine)	solute carrier family 47, member 1	0.645687	2.158	2.886
SLC47A2	Organic cations (e.g. creatinine)	solute carrier family 47, member 2	0.60417	3.179	2.282
SLCO2A1	Prostaglandins	solute carrier organic anion transporter family, member 2A1	0.000609	70.11	123.911*
SLCO2B1	Steroids, prostaglandins	solute carrier organic anion transporter family, member 2B1	0.004457	3.665*	2.195
SLCO4C1	thyroxin	solute carrier organic anion transporter family, member 4C1	1.95E-05	68.57*	36.456

*Asterisk indicates greater expression in cell type indicated (P<0.05).

One final caveat regarding these results is that although SLC genes are all involved in solute transport across membranes, not all are involved in nutrient transport across the plasma membrane, which would be required for a role in transport between the dam and fetus. Many are involved in transport between organelles within cells (e.g., SLC25 genes are all mitochondrial transport genes). Some provide substrate influx into the cell, while others provide substrate efflux, and some do both depending on their cellular location (e.g., apical vs. basal) and the conditions in and around the cell. Because two epithelial cell layers separate maternal and fetal blood in the pig, all nutrients must pass four intact cell membranes to reach the fetal blood, so whether they participate in influx or efflux, they would still contribute to nutrient transport to the fetus. Despite these limitations, the results provide suggestions for future experiments that could be directed at understanding the transport of nutrients and the physiology of the pig placenta.

### General

Of the 293 SLC genes in the Swine Genome build 9.2, 108 genes (37% of total) had expression values greater than 1.8 PKM. Of these, the expression of 30 (28% of expressed) were greater in short cuboidal compared to tall columnar cells, and 10 (9% of expressed) were greater in tall columnar cells compared to short cuboidal cells. This compares to 20,461 genes in Swine Genome build 9.2; 7,413 (36% of total) genes expressed by trophoblast cells, and 1,088 genes (15% of expressed) greater in short cuboidal and 434 genes (6% of expressed) greater in tall columnar cells. Thus, transport genes do not appear to be preferentially expressed by trophoblast cells as a group compared to all genes, but preferential expression of SLC genes by short cuboidal cells appears to be greater generally than tall columnar cells, and greater than the frequency of preferential expression of all genes by short cuboidal cells. This may suggest that nutrient transport occurs preferentially through short cubiodal trophoblast cells, which is also consistent with their small size and greater surface area relative to tall columnar cells.

### Sugars

Sugars are polar molecules, and their transport is mediated by specific proteins, facilitative glucose transporters (GLUT) and Na-dependent glucose transporters (SGLT), which are involved in passive and active transport of glucose, respectively [54]. SLC2 and SLC5 genes, respectively, correspond to these two types of transporters [55, 56]. Sequences matching SLC2A7 were not observed. The two most highly expressed SLC2 genes were SLC2A12 and 13, corresponding to GLUT12 and proton-dependent inositol transporter, respectively. SLC2A4 and 10 (GLUT4 and 10) were also observed. SLC2A12 expression was greater in tall columnar cells compared to cuboidal cells, while the reverse was true for SLC2A13. GLUT12 and GLUT4 are insulin dependent glucose transporters [57], but levels of insulin are reported to be very low in the pig fetus [39]. However, insulin like growth factors (IGF) can also regulate GLUT4 transporters [58] (similar results are not available for GLUT12), suggesting that IGF1 and 2 may provide regulation of glucose transport to the pig conceptus via changes in GLUT4 and GLUT12. Also, the high expression and increased presence of GLUT12 in tall columnar trophoblast cells suggest that these cells may be specialized to provide a regulatory role for placental function through regulation of glucose transport.

The second highest SLC2 gene expressed by placenta, SLC2A13, corresponds to proton-dependent inositol transporter, which is an inositol-H^+^ cotransporter that follows a proton gradient and therefore represents active transport for inositol [59, 60]. The high expression of this active transporter suggests (1) that inositol has an important role in some aspect of conceptus development or function and (2) that transport of inositol would be encouraged by acidic conditions within the placenta. Consistent with an important role for inositol, concentrations in the fetus are unusually high (4-5 mmol/L, rivaling fructose), and are greater in small fetuses compared to large fetuses [61, 62]. Phospholipids containing inositol, and inositol phosphates derived from them, are second messengers for a variety of receptor systems [63]. Inositol containing lipids also participate in cell membrane fusions required for trafficking of cell membrane components [64]. In addition, many membrane proteins are anchored to the cell membrane through glycophosphatidylinositol linkages [65]. Although these second messenger systems and other biochemical processes that require inositol are vital, they are unlikely to require transport of large amounts and high concentrations of inositol. Inositol can also be converted to glucuronic acid through the action of myo-inositol oxygenase (MIOX) [66]. Sequences matching the MIOX gene were found in trophoblast cells, and were greater in the short cuboidal cells similar to the SLC2A13 sequences. Glucuronic acid is a component of hyaluronan, but mammals like the pig apparently lack the enzymes necessary to generate UDP-glucuronate from glucuronic acid [67], instead UDP-glucuronate is generated using UDP-glucose as a substrate. Free glucuronic acid can be metabolized to CO_2_ in the pentose shunt pathway, so it could be used for energy. Alternatively, glucuronate is also a substrate for the generation of ascorbic acid [68]. Ascorbic acid is one of the main antioxidants available to tissues to prevent oxidative damage from free radicals. Ascorbic acid is also required to transfer the iron contained in uteroferrin to transferrin [69]. Whether inositol is used for inositol lipids and second messengers, glucuronic acid, ascorbic acid or energy generation in conceptus development will require further study.

### Amino acids

A number of SLC genes are known transporters of amino acids including members of the SLC1, SLC3, SLC6, SLC7, SLC16, SLC36 and SLC38 gene families [70–76] (Table 1). Of these, the three most highly expressed were SLC1A3, SLC7A4 and SLC16A10. The SLC1A3 gene codes for a glutamate/aspartate transport protein [74] (GLAST). In the brain, this protein is responsible for removal of glutamate, a potent neurotransmitter, from the synaptic space of glutamate releasing neurons. Glutamate is taken up by astrocytes and rapidly converted to glutamine, which is then released back to the neurons for reuptake and synthesis of glutamate. Interestingly, our results indicate that glutamine synthetase is highly expressed by both types of trophoblast cells (GLUL, short cuboidal cells 328.9 FPKM, tall columnar cells 388.2 FPKM), suggesting that some portion of glutamate transported into cells is likely to be converted to glutamine. Consistent with this, the glutamine concentration in fetal plasma was greatest of all the amino acids throughout most of gestation, and was much higher than maternal plasma levels [77, 78]. As previously indicated, glutamine plays a role in glucosamine synthesis along with fructose, providing substrate for hyaluronan and other glycosamine glycans, and glucosamine synthesis is known to be tied to mTOR control of proliferation of day 12 trophoblast cells [44, 45]. Thus, it seems possible that glutamine and fructose combine to provide overall regulation of placental development, through generation of glycosaminoglycans and control of proliferation of trophoblast cells.

SLC7A4 codes for the CAT-4 protein, which is related to the other members of SLC7 family of cationic amino acid transporter. Proteins encoded by the SLC7A1, 2 and 3 genes are membrane proteins that transport lysine, arginine and ornithine, and correspond to the y+ amino acid transport system. However, the CAT-4 protein apparently has no intrinsic transporter activity [76], so its function is not clear. Supplemental arginine is known to increase litter size, possibly through its role in NO synthesis [79, 80]. However, because it is not clear what role the SLC7A4 gene has in cationic amino acid transport or metabolism, its role in arginine metabolism is also unclear.

The SLC16A10 gene encodes the MCT10 protein, an aromatic amino acid transporter [71]. Three of the four aromatic amino acids are nutritionally essential (histidine, tryptophan, phenylalanine), while the fourth (tyrosine) can be synthesized from phenyalanine. The high expression of this gene may be related to the fact that most of the transported amino acids cannot be synthesized by the fetus. In addition, the MCT10 protein is not Na+ or H+ coupled, so transport relies on the concentration gradient for these amino acids. The concentrations of all four amino acids in maternal and fetal plasma throughout gestation are consistent with passive transport as the mechanism of transport (fetal concentration is lower than maternal) [77]. If the high expression of the gene is related to the essential nature of the amino acids and the passivity of transport, it suggests that transport of these amino acids might be particularly sensitive to factors that impair SLC16A10 expression or mRNA translation, or factors that result in reduced concentrations of aromatic amino acids in the maternal blood (e.g. shortages in the diet).

Curiously, trophoblast cells highly express the SLC15A2 gene, which is a di/tripeptide transporter [81]. This suggests an alternative route of amino acid absorption by the placenta, via absorption of di- and tri-peptides, even though low levels of these are likely to be circulating in maternal blood. Alternatively, this transporter could function to allow transport of specific di- and tripeptides, such as carnosine and glutathione, respectively. Both provide antioxidant activity, which may be important in protecting the fetus from free radicals generated during respiration. Whether significant amino acids are transported to the placenta as di- and tri-peptides, or whether the SLC15A2 gene serves another role in conceptus metabolism will require further study.

### Lipids

Of the SLC genes found to be expressed by trophoblast cells, only SLC27A4 and SLC27A6 transport lipids in the form of long chain fatty acids [82, 83]. Neither of these genes is highly expressed by trophoblast cells (Table 1), which is consistent with previous reports indicating very poor transport of fatty acids by pig placenta [51]. Alternatively, lipids could be transported to the developing conceptus in the form of triglycerides associated with lipoproteins. Although they are not SLC genes, receptors for lipoprotein complexes are expressed by trophoblast cells, with the greatest being LRP6 (SC cells 10.9, TC cells 8.8 FPKM; McNeel et al., unpublished observations) [84]. Thus, it is likely that lipids reach the conceptus through two mechanisms, transport as free fatty acids, which appears likely to be poor, and as lipids associated with lipoproteins.

### Vitamins

Vitamins are transported by SLC5A6 (biotin, pantothenic acid) [85], SLC19A2 (thiamine) [86], SLC25A32 (mitochondrial folate) [87], SLC44A1, 2 and 3 (choline) and SLC44A4 (thiamine) [88] and SLC46A3 (folate) [86]. The two most highly expressed are SLC5A6 and SLC44A1. SLC5A6 is a multivitamin transporter, and the high expression is likely to be related to the essential nature of these vitamins as cofactors in metabolic reactions. High expression of SLC44A1 (and SLC44A2) suggests that high levels of choline are needed for some aspect of fetal development. Choline is a component of choline containing phospholipids (phosphatidylcholine and sphingomyelin), which themselves are components of cell membranes. Also, choline is a component of the neurotransmitter acetylcholine. Finally, choline is a source of methyl groups for methylation reactions [89]. Methylations are particularly important for placental function, as many imprinted genes that influence placental function are controlled by differential methylation of gene copies originating from either the sire or the dam [90].

### Minerals

Zinc is transported by SLC30A4, A6, A7, and A9, and SLC39A7, 8, 9, 11, and 14 [91–93]. The combined expression of these genes suggests that zinc is very important to the physiology and biochemistry of the fetus. Zinc participates in a variety of biochemical reactions, including antioxidant activity (superoxide dismutase) and prostaglandin synthesis [94–96]. But the most important to the fetus is likely to be its incorporation into carbonic anhydrase, which plays a role in metabolism of CO_2_[50]. Carbonic anhydrase catalyzes the formation of carbonate ion from CO_2_, and is a major mechanism enabling the safe transport in serum of CO2 away from tissues after it is produced. Interestingly, a recent study indicated that supplementation of zinc during late pregnancy reduced the incidence of stillbirths in low birth weight piglets, reinforcing the importance of zinc to the developing pig fetus [97].

Other minerals are also transported by SLC genes [87, 98–101], specifically iron (SLC25A28 and A37, both mitochondrial), copper (SLC31A2), sulfate (SLC13A4, SLC26 A2) and phosphate (SLC20A1, mitochondrial SLC25A3). Transport by these SLC genes likely support heme production for respiratory chain enzymes, copper containing superoxide dismutase and other copper containing proteins, sulfation of proteoglycans and organic compounds and myriad phosphorylation reactions.

### Hormones

Although not nutrients, transport of hormones in and out of trophoblast cells is likely to have important effects on placental function in an autocrine and paracrine manner, and on the fetus in an endocrine manner. SLC genes [102, 103] participate in transport of prostaglandins (SLCO21A1), steroids (SLC10A7, SLCO2B1) and thyroid hormones (SLCO4C1). Secretion of prostaglandins by the placenta are key regulators of the initiation of farrowing [104] and probably also participate in the control of blood flow. Placental tissues secrete large amounts of estrogen [105, 106], which likely plays a role in mammary gland development during pregnancy. The role of thyroid hormones in the placenta is less clear, but the expression level of the SLCO4C1 gene suggests that further work may be warranted to understand how thyroid hormones may regulate placental function or fetal development in pigs.

### Summary

The pig placenta mediates nutrient transport between the dam and the developing pig fetus. Physical characteristics and the expression of nutrient specific mechanisms of the placenta combine to determine the efficiency of transport of the various nutrients required for the development of a healthy piglet at birth. Further work is needed to understand and enhance the development of the folded epithelial bilayer of the pig placenta. Clues to important transport mechanisms for the pig placenta are found in the expression levels of various genes, but further work is needed to confirm the roles of these genes in transport and suggest dietary or management strategies that will improve the transport of specific nutrients to the developing pig fetus. It is still unclear what nutrients currently limit fetal development in the pig.

## Conclusions

We are only beginning to understand the physiological mechanisms that control the morphology and nutrient transport capability of the pig placenta, but it is likely that further understanding will allow not only improvements in litter size, but also reduction of stillbirth and preweaning mortality. Inefficiencies in placental function during gestation are likely to be a primary contributor to perinatal and neonatal piglet losses, as well as the number of fully formed fetuses at farrowing.

### Animal care and use

Previously unpublished experiments described in this report were approved by the USMARC Animal Care and Use committee, and conformed to FASS guidelines for use of Agricultural animals in research.

## Authors’ original submitted files for images

Below are the links to the authors’ original submitted files for images. Authors’ original file for figure 1 Authors’ original file for figure 2

## Abbreviations

GLAST: Glutamate/aspartate transport protein
GLUL: Glutamate-ammonia ligase
GLUT: Glucose transporters
IGF: Insulin like growth factors
MIOX: Myo-inositol oxygenase
FPKM: Fragments per kilobase of exon model mapped per million mapped reads
SC: Short cuboidal
SGLT: Na-dependent glucose transporters
SLC: Solute carrier
TC: Tall columnar
UHO: Unilateral hysterectomy-ovariectomy
USMARC: U.S. Meat Animal Research Center
VEGF: Vascular endothelial growth factor.
