# Supplementary figures and images for: Placental accommodations for transport and metabolism during intra-uterine crowding in pigs
Source: J Anim Sci Biotechnol. 2014 Dec 15;5:55. doi: 10.1186/2049-1891-5-55 (PMC4416243; doi:10.1186/2049-1891-5-55)

a.

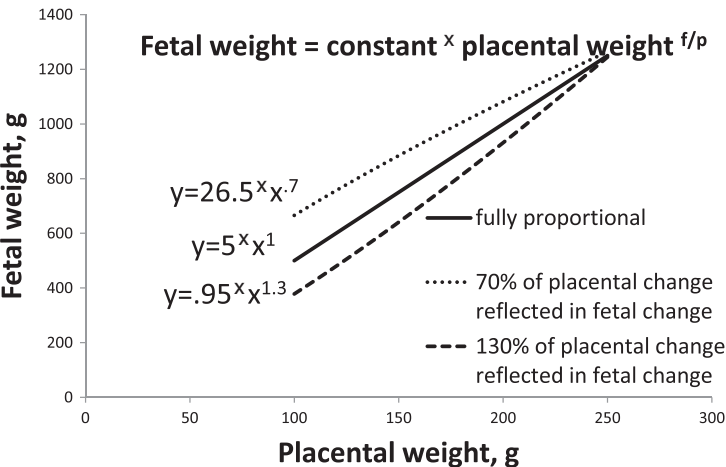

b.

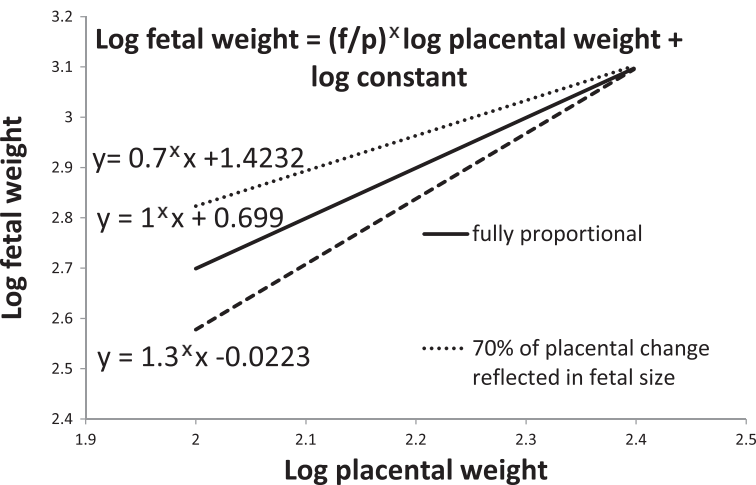

Supplement: Supplementary file 1 — Authors’ original file for figure 1 [file 40104_2014_150_MOESM1_ESM.pdf]

a.

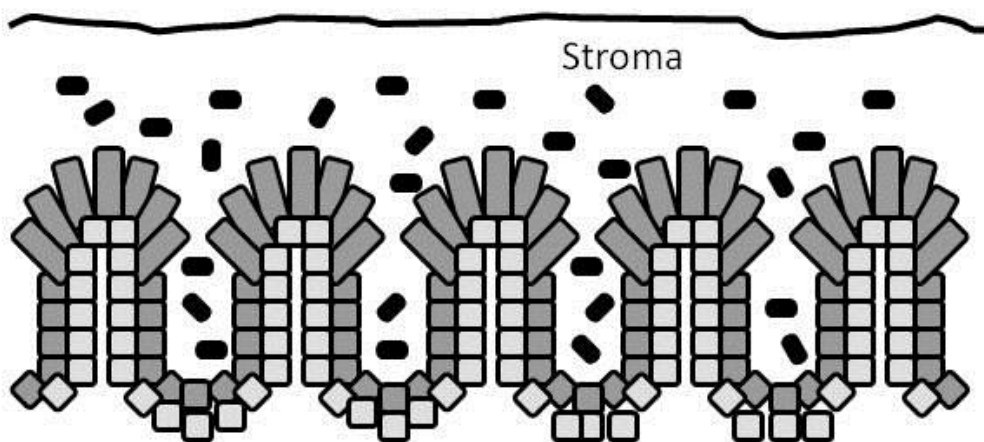

b.

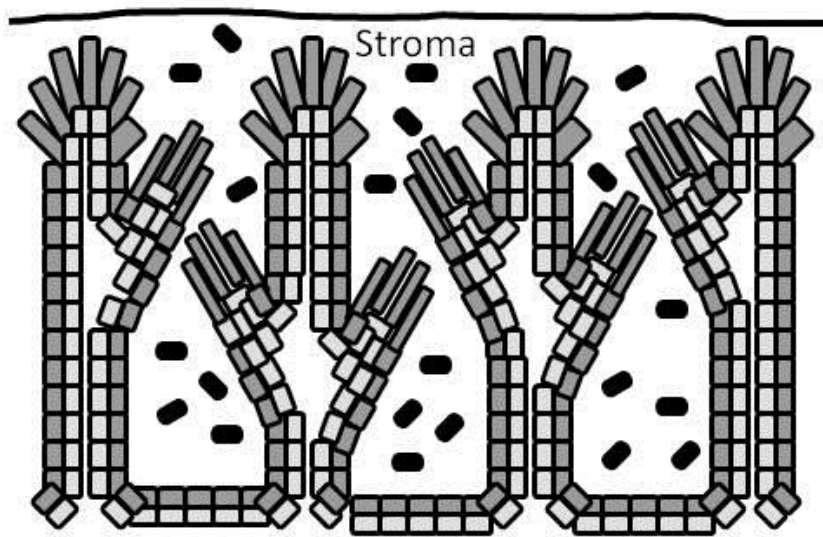

Supplement: Supplementary file 2 — Authors’ original file for figure 2 [file 40104_2014_150_MOESM2_ESM.pdf]
